# Immediate and Intermediate-Term Outcomes of Right Ventricular Outflow Tract Interventions in Neonates and Infants: A Prospective Single-Center Study

**DOI:** 10.7759/cureus.92516

**Published:** 2025-09-17

**Authors:** Bhavik Champaneri, Vicky Garhwal, Abhay Pota, Tarun Parmar, Shilpa Deodhar, Amit Kungwani

**Affiliations:** 1 Pediatric Cardiology, U.N. Mehta Institute of Cardiology and Research Centre, Ahmedabad, IND; 2 Cardiology Department, Geetanjali Medical College and Hospital, Udaipur, IND; 3 Anesthesia, U.N. Mehta Institute of Cardiology and Research Centre, Ahmedabad, IND

**Keywords:** balloon pulmonary valvotomy, patent ductus arteriosus stenting, pulmonary stenosis, right ventricular outflow tract, rvot perforation

## Abstract

Background: Right ventricular outflow tract (RVOT) anomalies in neonates and infants necessitate early intervention to restore adequate pulmonary blood flow and promote pulmonary artery (PA) growth. This study aimed to evaluate the immediate and intermediate-term outcomes of various transcatheter RVOT interventions in this vulnerable population.

Methods: This prospective, single-center descriptive study enrolled 52 infants (aged <1 year) undergoing balloon pulmonary valvotomy (BPV, n=29), RVOT perforation with BPV and patent ductus arteriosus (PDA) stenting (n=11), or RVOT stenting (n=12) between February 2021 and November 2022. Key immediate outcomes included hemodynamic changes, procedural success, and in-hospital complications. Intermediate outcomes at three and six months assessed oxygen saturation (SpO2), weight gain, PA growth, and re-intervention rates.

Results: Overall six-month survival was high at 96.2% (50/52), with minimal procedural complications, which included two transient arrhythmias. In-hospital mortalities occurred in one BPV patient and one RVOT perforation patient, both attributed to non-procedural causes such as septic shock.

BPV procedures achieved significant reductions in RV pressure (from 106±26.18 mmHg to 40.74±9.91 mmHg, p=0.04) and RVOT gradient (from 85.41±26.04 mmHg to 15.67±4.2 mmHg, p<0.0001), with a 14.3% re-intervention rate for restenosis by three months. For RVOT perforation with BPV and PDA stenting, there was marked hemodynamic improvement, with RV pressure decreasing from 105.36±18.98 mmHg to 40±12.06 mmHg (p<0.0001) and robust PA growth (RPA measuring 5.72±1.13 mm and LPA 5.10±0.93 mm at six months). Lastly, RVOT stenting was 100% successful, significantly improving SpO2 (from 73.83±5.46% to 86.83±4.61% at discharge) and enabling five infants to become candidates for complete intracardiac repair by six months.

Conclusion: Transcatheter RVOT interventions are safe and effective strategies for managing RVOT anomalies in neonates and infants. These intervention strategies offer targeted benefits and improve clinical outcomes.

## Introduction

Right ventricular outflow tract (RVOT) anomalies represent a diverse spectrum of congenital heart defects, frequently necessitating diverse interventional strategies. Neonates and infants, owing to their inherent physiological fragility, represent a particularly high-risk population for cardiac interventions, with age less than six months being a recognized risk factor for adverse outcomes [1]. We present prospective data from a single cardiac center, focusing on procedural outcomes and six-month follow-up of three distinct RVOT interventions. The high-risk demographic of our patients, notably a substantial proportion of neonates (72.73%) and low-birth-weight infants (54.55% <2.5 kg) within the RVOT perforation group, profoundly influenced the context of our interventions. Due to varied anatomy and evolving techniques, documenting early and mid-term outcomes is essential for guiding clinical decision-making and informing future research. While individual interventions have been extensively studied, revealing varied re-intervention rates and long-term considerations [2-4], comprehensive data describing the outcomes of a spectrum of RVOT interventions - including balloon pulmonary valvotomy, RVOT perforation with balloon pulmonary valvotomy (BPV) and patent ductus arteriosus (PDA) stenting, and RVOT stenting - in a single cohort of neonates and infants, with consistent intermediate-term follow-up, remains vital. This study aimed to describe the immediate and three- and six-month intermediate-term outcomes, encompassing safety, efficacy, and pulmonary artery growth, of various transcatheter RVOT interventions performed in a cohort of infants at our tertiary cardiac care institution.

## Materials and methods

Study design and ethical approval

This was a prospective, single-center descriptive study conducted at our tertiary cardiac care institute from February 2021 to November 2022. Ethical approval for the study was obtained from the institutional ethics committee (UNMICRC/CARDIO/2021/01).

Patient population

Infants aged up to one year (i.e., <365 days) requiring transcatheter interventions on the RVOT were included. Patients older than one year at the time of intervention were excluded. Our study cohort comprised patients undergoing BPV, RVOT perforation for membranous pulmonary atresia with BPV with or without PDA stenting, and RVOT stenting.

Data collection

Baseline clinical assessment encompassed demographic details (age, weight, gender), 12-lead surface electrocardiogram (ECG), echocardiography, and cardiac catheterization with angiography. During the procedure, we collected data on the type and size of catheters, wires (e.g., chronic total occlusion (CTO) wires: Gaia, Fielder XT, Cross IT 200, all Asahi Intecc, Seto, Japan), balloon sizes and annulus-to-inflation ratios, stent types (e.g., Resolute Onyx, Medtronic, Minneapolis, MN, USA; Omnilink Elite, Abbott, Chicago, IL, USA), fluoroscopy time, procedure time, contrast dye volume (standardized at 4 ml/kg), and immediate complications. Patients were followed up at three months and six months post-procedure. At each follow-up visit, oxygen saturation (SpO2) and weight were measured. Echocardiography was performed to assess right ventricular (RV) function, residual pulmonary stenosis (PS), degree of pulmonary regurgitation (PR), and right and left pulmonary artery (RPA and LPA) sizes.

Echocardiogram was done with S8-3 and S12-4 pediatric echocardiography probes. RVOT anatomy was defined in parasternal short axis view for BPV and RVOT perforation, ductal anatomy and branch PA anatomy was assessed in parasternal short axis, suprasternal short axis and suprasternal long axis views for PDA stenting. Besides, subcostal enface view was used to define RVOT length and main pulmonary artery (MPA) diameter for RVOT stenting.

Interventional procedures

All procedures were generally conducted under intravenous sedation. Endotracheal ventilation was selectively employed in high-risk patients already receiving mechanical ventilation or high inotropic support. The percutaneous femoral venous route served as the primary access choice, with jugular venous access as an alternative. All procedures were performed by a single operator.

For BPV, we measured RV and PA pressures and performed angiography to confirm the obstruction site, RV size, and pulmonary valve annulus. A soft J-tipped wire and a 4F or 5F right coronary artery (RCA) catheter were advanced across the pulmonary valve into the distal pulonary artery. Subsequently, a 0.018-inch steel core stiff wire was exchanged. Balloon selection typically involved a size 2 mm larger than the pulmonary annulus. The balloon was then inflated manually with diluted contrast until the waist disappeared.

In RVOT perforation with BPV and PDA stenting procedures, patients with unfavorable anatomy (e.g., small/absent RVOT, increased RVOT-MPA distance, no discrete plate-like valve) were excluded. Femoral venous and arterial access (4F/5F short sheaths) was obtained. A 4F JR catheter was positioned in the RVOT, and a 4F angled glide catheter in the PDA mouth. A 0.014 BMW wire (Abbott) was advanced across the PDA into the MPA as a target. A second CTO wire (Crossit 200, Fielder XT, Gaia 2) was then utilized to perforate the plate-like valve from the RVOT. The wire was snared from the MPA via the arterial catheter to create an A-V loop. A balloon catheter subsequently dilated the perforated valve. A 4F, 45-cm-long sheath was positioned in the MPA. PDA stenting was selectively performed in cases with persistent desaturation, with stent selection guided by echocardiographic and angiographic measurements of the PDA.

For RVOT Stenting, Resolute Onyx (4.5 mm, 5 mm) and Omnilink (6 mm) stents were deployed. Stent sizing aimed for 1-2 mm larger than the MPA diameter, carefully avoiding coronary artery overstretching and encroachment on branch PAs. Stent length ensured adequate coverage of the RVOT, pulmonary valve, and distal MPA. Normal coronary artery anatomy was confirmed in all patients prior to stenting.

Anticoagulation Protocol

Heparin was administered intra-procedurally to maintain an activated clotting time (ACT) of 190-220 seconds. Post-procedure, heparin infusion continued at 10 IU/kg/hour for 24 hours (activated partial thromboplastin time (aPTT) target 1.5-2 times normal). Patients were then transitioned to aspirin and clopidogrel. Prophylactic antibiotics were administered to all patients.

Our antiplatelet protocol was to give 10 mg/kg aspirin night before procedure and then give 5mg/kg aspirin once a day and 0.5-1 mg/kg/dose clopidogrel twice a day post procedure in those who underwent PDA stenting while only aspirin in those who underwent RVOT stenting till next palliative/corrective procedure or for at least one year in those with RVOT perforation + BPV + PDA stenting who did not require any further procedures. In addition, we give beta-blocker therapy in RVOT stenting and RVOT perforation patients.

Definitions

Procedural Success

Procedural success for BPV was defined as a post-procedure pulmonary gradient of <40 mmHg. For RVOT perforation + BPV + PDA stenting, it was defined as the establishment of reliable antegrade pulmonary blood flow, at least mild PR, successful stent deployment, and SpO2 > 85%. For RVOT stenting, procedural success was defined as successful stent deployment across the RVOT with SpO2 >85%.

Missing Data

No significant missing data were encountered that would impact the study's conclusions.

Statistical analysis

All statistical analyses were performed using SPSS program version 20 (IBM Corp., Armonk, NY, USA). Quantitative variables were expressed as the mean ± standard deviation. Qualitative variables were expressed as percentages (%). Comparison of parametric values between two groups was performed using the independent sample t-test. Categorical variables were compared using the chi-square test. A two-tailed p-value <0.05 was considered statistically significant.

## Results

A total of 52 infants underwent RVOT interventions during the study period. The cohort was divided into three distinct groups: BPV, RVOT perforation with BPV and PDA stenting, and RVOT stenting.

Patient demographics and baseline characteristics

The baseline characteristics of our study population are summarized in Table 1. Median age varied across groups: 88 days (range: 1-340 days) for BPV, 45 days (range: 1-90 days) for RVOT perforation + BPV + PDA stenting, and 63 days (range: 5-340 days) for RVOT stenting. Notably, 72.73% of the RVOT perforation group were neonates (0-30 days old). Mean weight was lowest in the RVOT perforation group (2.67±0.69 kg), with 54.55% weighing <2.5 kg, reflecting a higher proportion of preterm or low-birth-weight infants in this subgroup. Pre-procedure SpO2 was lowest in the RVOT stenting group (73.83±5.46%), followed by RVOT perforation (81.09±11.16%) and BPV (86.17±12.66%). Hemoglobin levels were commensurately higher in the cyanotic groups. Isolated PS (doming or dysplastic) was the predominant diagnosis in the BPV group, accounting for 75.86% of cases. Two patients in the BPV group (6.9%) also presented with pulmonary stenosis with PDA. The RVOT perforation group exclusively comprised pulmonary atresia with intact ventricular septum (PA-IVS, 100%). The RVOT stenting group primarily included patients with tetralogy of Fallot (TOF, 75%) and double-outlet right ventricle (DORV) with TOF physiology (25%).

**Table 1:** Baseline Characteristics BPV: balloon pulmonary valvotomy, RVOT: right ventricular outflow tract, PDA: patent ductus arteriosus, PS: pulmonary stenosis, PA-IVS: pulmonary atresia with intact ventricular septum, TOF: tetralogy of Fallot, DORV: double-outlet right ventricle

Characteristic	BPV (N=29)	RVOT perforation + BPV + PDA stenting (N=11)	RVOT stenting (N=12)
Sex: Female (%)	14 (48.3%)	05 (45.5%)	03 (25%)
Male (%)	15 (51.7%)	06 (54.5%)	09 (75%)
Median age (days) (Range)	88 (1-340)	45 (1-90)	63 (5-340)
Age 0-30 days (%)	11 (37.93%)	8 (72.73%)	7 (58.33%)
Age 30-60 days (%)	1 (3.45%)	01 (9.09%)	2 (16.67%)
Age >60 days (%)	17 (58.62%)	2 (18.18%)	3 (25%)
Mean Weight (kg) (± SD)	4.29 ± 1.84	2.67 ± 0.69	3.97 ± 1.79
Weight < 2.5 kg (%)	4 (13.79%)	6 (54.55%)	1 (8.33%)
Height (± SD)	54.90 ± 9.28	46.73 ± 4.05	56.58 ± 17.86
Preterm			
Mean Heart rate (bpm) (± SD)	137.10 ± 18.60	148.55 ± 18.41	147.25 ± 23.97
Mean SpO2 (%) (± SD)	86.17 ± 12.66	81.09 ± 11.16	73.83 ± 5.46
Mean Haemoglobin (g/dL) (± SD)	14.46 ± 3.28	16.84 ± 2.85	16.34 ± 1.06
Primary Diagnosis (%)			
Isolated PS (Doming)	17 (58.62%)	0	0
Associated supravalvar PS	4 (13.79%)	0	0
Isolated PS (Dysplastic)	05 (17.24%)	0	0
Pulmonary stenosis with PDA	02 (6.9%)	0	0
PA-IVS	0	11 (100%)	0
TOF	03 (10.3%)	0	09 (75%)
DORV (TOF)	02 (6.8%)	0	03 (25%)

Pre-procedure echocardiographic parameters are presented in Table 2. The pulmonary artery annulus was largest in the BPV group (7.06±1.84 mm). RV morphology was predominantly tripartite in BPV (93.1%) and RVOT stenting (100%) groups, while bipartite RVs were more common in the RVOT perforation group (63.6%). Overall, the cohort included nine bipartite and 43 tripartite RVs. None of the patients in the RVOT perforation group had right ventricle-dependent coronary circulation (RVDCC) as ruled out by RV and aortic root angiography.

**Table 2:** Pre-procedure Echocardiographic Parameters RVOT: right ventricular outflow tract, BPV: balloon pulmonary valvotomy, RPA: right pulmonary artery, LPA: left pulmonary artery, TV: tricuspid valve, RV: right ventricle

Parameter	BPV (N=29)	RVOT perforation + BPV + PDA stenting (N=11)	RVOT stenting (N=12)
PA annulus (mm) (± SD)	7.06 ± 1.84	5.63 ± 0.64	5.6 ± 0.83
RPA (mm) (± SD)	4.33 ± 0.65	3.83 ± 0.52	3.98 ± 0.91
LPA (mm) (± SD)	3.86 ± 0.54	3.83 ± 0.42	3.67 ± 1.07
TV annulus (mm) (± SD)	10.45 ± 1.50	8.49 ± 2.05	9 ± 1.41
RV size (%)			
Dilated	12 (41.4%)	0	0
Hypertrophied	01 (3.4%)	0	0
Hypoplastic	07 (24.1%)	11 (100%)	01 (8.3%)
Normal	09 (30%)	0	11 (91.7%)
RV type (%)			
Bipartite	02 (6.9%)	07 (63.6%)	0
Tripartite	27 (93.1%)	04 (36.4%)	12 (100%)

Immediate procedural outcomes

All procedures were performed via femoral vascular access under intravenous sedation, with selective endotracheal ventilation employed in three high-risk cases.

Group A: Balloon Pulmonary Valvotomy

For the 29 patients in the BPV group, the mean procedure time was 15.1 minutes, with a mean fluoroscopy time of 11.8 minutes. Tyshak balloons were utilized in a majority of the procedures. Immediate post-procedure hemodynamic changes are detailed in Table 3. Mean RV pressure significantly decreased from 106±26.18 mmHg to 40.74±9.91 mmHg (p=0.04), and the RVOT gradient notably reduced from 85.41±26.04 mmHg to 15.67±4.2 mmHg (p=<0.0001). SpO2 improved from 86.17±12.66% pre-procedure to 94.71±5.27% at discharge. The procedural success rate stood at 96.55%.

**Table 3:** Immediate Hemodynamic Outcomes BPV: balloon pulmonary valvotomy, RVOT: right ventricular outflow tract, PDA: patent ductus arteriosus, RV: right ventricular. Paired t-test was used to calculate the difference between means. P value ≤0.05 shows statistically significant difference

Parameter	Time Point	BPV (N=29)	t-statistic	p-value	RVOT perforation + BPV + PDA stenting (N=11)	t-statistic	p-value	RVOT stenting (N=12)	t-statistic	p-value
Mean RV pressure (mmHg) ± SD	Pre-procedure	106 ± 26.18	-12.55	<0.0001	105.36 ± 18.98	-65.36	<0.0001	94.29 ± 26.01	-0.93	0.36
	Post-procedure	40.74 ± 9.91			40 ± 12.06			88.29 ± 23.03		
Mean Gradient (mmHg) ± SD	Pre-procedure	85.41 ± 26.04	-14.24	<0.0001	96.55 ± 20.30	-72.55	<0.0001	85.25 ± 21.96	-0.37	0.82
	Post-procedure	15.67 ± 4.20			24 ± 2.20			83.69 ± 6.52		
Mean SpO₂ (%) ± SD	Pre-procedure	86.17 ± 12.66	3.35	0.001	81.09 ± 11.16	8.82	0.0003	73.83 ± 5.46	9.80	<0.0001
	Post-procedure	94.71 ± 5.27			89.91 ± 5.26			86.83 ± 4.61		
Procedural Success Rate (%)	Immediate	96.55%		–	81.8%		–	100%		

In-hospital outcomes are summarized in Table 4. The mean ICU stay was one day, and mean hospital stay was 4.3 days. At discharge, PR was observed as: no PR in 17 (58.62%), mild in eight (27.5%), moderate in two (6.8%), and severe in one (3.4%). Residual PS was absent in 16 (57.14%), mild in seven (25%), moderate in one (3.5%), and severe in four (14.2%). Transient arrhythmias were developed in two patients in this group during the procedure, which resolved spontaneously. In-hospital mortality was observed in one patient with this group, attributed to septic shock.

Intermediate-term follow-up results at three and six months, patients maintained excellent SpO2: 95.64±4.65% at three months and 96.11±4.04% at six months. Consistent weight gain was observed: 5.75±1.82 kg at three months and 7.42±1.56 kg at six months. RPA diameter increased to 4.84±0.50 mm (three months) and 5.03±0.49 mm (six months), and LPA diameter similarly increased to 4.23±0.50 mm (three months) and 4.38±0.35 mm (six months). PS was absent in 16 (57.14%) at three months and 18 (64.2%) at six months, with mild PS present in seven (25%) at three months and eight (28.5%) at six months. RV function remained normal in 27 (93.1%) at both follow-ups, with dysfunction in two (6.9%). The re-intervention rate was four (14.3%) at three months, primarily for restenosis requiring redo BPV.

Group B: RVOT Perforation With BPV and PDA Stenting

For the 11 patients in the RVOT perforation + BPV + PDA stenting group, the mean procedure time was 29.5 minutes, with a mean fluoroscopy time of 25.2 minutes. Immediate post-procedure hemodynamic changes are detailed in Table 3. Mean RV pressure dropped substantially from 105.36±18.98 mmHg to 40±12.06 mmHg (p=<0.0001), and the gradient also significantly decreased from 96.55±20.3 mmHg to 24±2.2 mmHg (p=<0.0001). SpO2 rose from 81.09±11.16% to 89.91±5.26% at discharge. Procedural success was achieved in 81.8% of cases. In-hospital outcomes are summarized in Table 4. Pre-operative mechanical ventilation and inotropic support were required in three cases each. The mean ICU stay was six days, and mean hospital stay was 10.3 days. At discharge, PR was observed as: no PR in one (10%), and mild in nine (90%). No moderate or severe cases were noted. Residual PS was absent in one (10%), mild in seven (70%), moderate in one (10%), and severe in one (10%). In-hospital mortality occurred in one patient within this group, in a neonate (< 30 days old) and was attributed to septic shock.

**Table 4:** Procedural and In-Hospital Outcomes at Discharge BPV: balloon pulmonary valvotomy, RVOT: right ventricular outflow tract, PDA: patent ductus arteriosus, PS: pulmonary stenosis, PR: pulmonary regurgitation

Parameter	BPV (N=29)	RVOT perforation + BPV + PDA stenting (N=11)	RVOT stenting (N=12)
Mean Total Fluoroscopic Time (min)	11.8	25.2	11.5
Mean Total Procedure Time (min)	15.1	29.5	18.1
Pre-op Mechanical Ventilation (n)	0	3	0
Pre-op Inotropic Support (n)	0	3	0
Total ICU Stay (mean, days)	1	6	1
Total Hospital Stay (mean, days)	4.3	10.3	7.8
Pulmonary Regurgitation at Discharge (%)			
No PR	17 (58.62%)	01 (10%)	0
Mild PR	08 (27.5%)	09 (90%)	0
Moderate PR	02 (6.8%)	0	09 (75%)
Severe PR	01 (3.4%)	0	03 (25%)
Residual PS at Discharge (%)			
No PS	16 (57.14%)	01 (10%)	-
Mild PS	07 (25%)	07 (70%)	-
Moderate PS	01 (3.5%)	1 (10%)	-
Severe PS	04 (14.2%)	01 (10%)	-
In-Hospital Mortality (n)	1	1	0
Transient Arrhythmias	2	0	0

Intermediate-term follow-up results at three and six months’ modest improvement in SpO2 was noted: 89.50±5.54% at three months and 90.89±4.62% at six months. Significant weight gain was achieved: 4.61±0.89 kg at three months and 6.73±1.25 kg at six months. RPA diameter increased to 5.08±0.95 mm (three months) and 5.72±1.13 mm (six months), and LPA diameter similarly increased to 4.55±0.71 mm (three months) and 5.10±0.93 mm (six months). Mild PS persisted in seven (70%) at both three and six months. PR was predominantly mild (n=9) at both three and six months, with no severe cases. RV function remained normal in 10 (100%) at both follow-ups. Pulsatile Glenn shunt was required in two patients due to inadequate RV growth.

Group C: RVOT Stenting

For the 12 patients in the RVOT stenting group, the mean procedure time was 18.1 minutes, with a mean fluoroscopy time of 11.5 minutes. Resolute Onyx stents and one Omnilink stent (6 mm) were deployed. Immediate post-procedure hemodynamic changes are detailed in Table 3. As a palliative procedure, this group exhibited minimal hemodynamic change, aiming to prevent acute pulmonary edema. RV pressure shifted from 94.29±26.01 mmHg to 88.29±23.03 mmHg (p=0.36), and the gradient remained stable (85.25±21.96 mmHg to 83.69±6.52 mmHg, p=0.82). SpO2 improved from 73.83±5.46% to 86.83±4.61% at discharge, reflecting enhanced pulmonary blood flow. This group achieved a 100% procedural success rate.

In-hospital outcomes are summarized in Table 4. The mean ICU stay was one day, and mean hospital stay was 7.8 days. At discharge, PR was noted: moderate PR in nine (75%) and severe in three (25%). Residual PS was not applicable due to the nature of the intervention. No in-hospital mortalities were observed in this group.

Intermediate-term follow-up results at three and six months of all groups were mentioned in Tables 5, 6, respectively. SpO2 remained stable: 86.82±4.20% at three months and 87.55±3.93% at six months. Substantial weight gain was observed: 5.57±1.79 kg at three months and 7.39±1.1 kg at six months. This group exhibited robust PA growth: RPA 5.08±1.21 mm (three months) and 6.12±1.57 mm (six months), and LPA 4.50±1.17 mm (three months) and 5.05±1.3 mm (six months). Significant PR was noted: moderate in nine (75%) to severe in three (25%) at both time points. RV function remained normal in 12 (100%). No re-interventions were required at three months. By six months, five patients became candidates for complete intracardiac repair, with some requiring branch PA plasty (three RPA, one LPA).

**Table 5:** Intermediate-Term Clinical and Echocardiographic Outcomes at Three Months BPV: balloon pulmonary valvotomy, RVOT: right ventricular outflow tract, PDA: patent ductus arteriosus, PS: pulmonary stenosis, PR: pulmonary regurgitation, RPA: right pulmonary artery, LPA: left pulmonary artery, TV: tricuspid valve, RV: right ventricle

Parameter	BPV (N=28)	RVOT Perforation + BPV + PDA stenting (N=10)	RVOT Stenting (N=12)
SpO2 (± SD)	95.64 ± 4.65	89.50 ± 5.54	86.82 ± 4.20
Weight (kg) (± SD)	5.75 ± 1.82	4.61 ± 0.89	5.57 ± 1.79
RPA (mm) (± SD)	4.84 ± 0.50	5.08 ± 0.95	5.08 ± 1.21
LPA (mm) (± SD)	4.23 ± 0.50	4.55 ± 0.71	4.50 ± 1.17
Gradient (mmHg) (± SD)	16.74 ± 5.91	24.45 ± 3.59	84.09 ± 4.57
Residual PS (%)			
No PS	16 (57.14%)	01 (10%)	-
Mild PS	07 (25%)	07 (70%)	-
Moderate PS	01 (3.5%)	1 (10%)	-
Severe PS	04 (14.2%)	01 (10%)	-
Pulmonary Regurgitation (%)			
No PR	17 (60.71%)	01 (10%)	0
Mild PR	08 (28.57%)	09 (90%)	0
Moderate PR	02 (7.14%)	0	09 (75%)
Severe PR	01 (3.57%)	0	03 (25%)
RV Function (%)			
No RV dysfunction	27 (96.4%)	10 (100%)	12 (100%)
RV dysfunction	01 (3.6%)	0	0
Re-intervention Rate (%)	14.3%	0%	0%

**Table 6:** Intermediate-Term Clinical and Echocardiographic Outcomes at Six Months BPV: balloon pulmonary valvotomy, RVOT: right ventricular outflow tract, PDA: patent ductus arteriosus, PS: pulmonary stenosis, PR: pulmonary regurgitation, RPA: right pulmonary artery, LPA: left pulmonary artery, TV: tricuspid valve, RV: right ventricle

Parameter	BPV (N=28)	RVOT Perforation + BPV + PDA stenting (N=10)	RVOT Stenting (N=12)
SpO2 (± SD)	96.11 ± 4.04	90.89 ± 4.62	87.55 ± 3.93
Weight (kg) (± SD)	7.42 ± 1.56	6.73 ± 1.25	7.39 ± 1.1
RPA (mm) (± SD)	5.03 ± 0.49	5.72 ± 1.13	6.12 ± 1.57
LPA (mm) (± SD)	4.38 ± 0.35	5.10 ± 0.93	5.05 ± 1.3
Gradient (mmHg) (± SD)	18.93 ± 3.15	25.02 ± 2.14	85.92 ± 21.91
Residual PS (%)			
No PS	18 (64.28%)	01 (10%)	-
Mild PS	08 (28.57%)	07 (70%)	-
Moderate PS	02 (7.14%)	1 (10%)	-
Severe PS	0	0	-
Pulmonary Regurgitation (%)			
No PR	17 (60.71%)	02 (20%)	0
Mild PR	08 (28.57%)	08 (80%)	0
Moderate PR	02 (7.14%)	0	09 (75%)
Severe PR	01 (3.57%)	0	03 (25%)
RV Function (%)			
No RV dysfunction	27 (96.4%)	10 (100%)	12 (100%)
RV dysfunction	01 (3.6%)	0	0
Corrective surgery candidates (n)	0	0	5
Palliative Glenn shunt (n)	0	2	0
Branch PA plasty (n)	0	0	4 (3 RPA, 1 LPA)

## Discussion

RVOT anomalies in neonates and infants represent a diverse spectrum of congenital heart defects, requiring individualized transcatheter strategies. This study presents outcomes from a single-center experience involving three interventional techniques: BPV, RVOT perforation with BPV and PDA stenting, and RVOT stenting.

In patients with isolated pulmonary valvular stenosis, BPV demonstrated a high procedural success rate of 96.6%, along with substantial immediate hemodynamic improvements. Specifically, right ventricular pressure declined from 106±26.18 mmHg to 40.74±9.91 mmHg, and RVOT gradient dropped from 85.41±26.04 mmHg to 15.67±4.2 mmHg. These results are consistent with earlier studies by Luo et al. and Schmaltz et al., which support BPV as a safe and effective intervention [2,3]. Our observed re-intervention rate of 14.3% at six months, higher than the 9.8% reported by AlAkhfash et al. and Loureiro et al., could be attributed to factors such as younger patient age and a greater proportion of dysplastic valves (17.2%) [4,5]. Four of five patients with dysplastic valves developed severe residual stenosis, emphasizing the anatomical limitations of BPV in such cases and the need for tailored follow-up. Pulmonary regurgitation following BPV was minimal, with severe PR observed in only 3.6% of patients at six months. While reassuring in the short term, long-term monitoring remains important due to the risk of RV dilation and dysfunction associated with PR [6].

In neonates with PA-IVS, RVOT perforation with BPV and PDA stenting achieved procedural success in 81.8% of cases. This technique resulted in improved oxygenation and branch pulmonary artery growth, with mean RPA and LPA diameters reaching 5.72±1.13 mm and 5.10±0.93 mm, respectively, at six months. The use of CTO wires played a critical role in optimizing procedural success and safety, consistent with the findings of Sadiq et al. [7]. All patients required adjunct PDA stenting to ensure adequate oxygen delivery, particularly in those with bipartite or hypoplastic right ventricles.

Despite these promising outcomes, two patients required Glenn shunts due to limited RV growth [8]. This reinforces the anatomical challenges in achieving biventricular repair in PA-IVS. Literature such as the study by Wright et al. demonstrates that even with successful decompression, biventricular circulation may not always be feasible, and a staged approach may be necessary [9]. The evolution from transfemoral to transjugular approaches in neonatal transcatheter pulmonary valve perforation has also been described [10].

In patients with TOF or DORV with TOF physiology, RVOT stenting achieved 100% procedural success and notable improvements in oxygen saturation (from 73.83±5.46% to 86.83±4.61% at discharge). At six months, these patients exhibited robust pulmonary artery growth (RPA: 6.12±1.57 mm; LPA: 5.05±1.3 mm), facilitating definitive intracardiac repair in five cases. These findings align with prior studies by Quandt et al. and Sandoval et al., confirming the role of RVOT stenting as an effective bridge to surgery [11,12]. However, moderate to severe PR was universal in this group, necessitating careful follow-up and potential future intervention.

Across all groups, the overall survival rate was 96.2%, with two in-hospital deaths (one each in the BPV and RVOT perforation groups), both due to septic shock. The low complication rates, including only two transient arrhythmias, further affirm the procedural safety in a specialized setting.

Limitations

This study presents several limitations. First, its single-center, descriptive design, while offering detailed insights into our institutional experience, may inherently limit the generalizability of findings to other populations or settings with differing expertise or resources, due to potential variations in patient selection, procedural techniques, and post-procedural management across different institutions. Second, the six-month follow-up period, while sufficient for intermediate-term outcomes, is relatively short. Longer-term sequelae, such as stent stenosis, fracture, or the sustained success of subsequent surgical repairs, particularly in TOF patients, remain unexplored. Third, the absence of a comparative arm, such as the modified Blalock-Taussig shunt (mBTS), precludes a direct evaluation of RVOT interventions against surgical alternatives. Also, all interventions were performed by a single operator, potentially limiting reproducibility of outcomes in centers with varied operator experience.

Future research

Future research should prioritize multicenter studies with larger cohorts and extended follow-up periods (beyond six months) to evaluate critical long-term outcomes such as stent durability, PR progression, and the success of definitive surgical repairs, particularly in TOF and PA-IVS patients. Comparative studies, ideally randomized controlled trials where ethically feasible, are needed to directly assess RVOT interventions against surgical alternatives like the mBTS, thereby enhancing the generalizability of findings and informing optimal management strategies. Further investigation into the factors predicting RV growth and biventricular repair candidacy in PA-IVS patients post-perforation is also warranted.

## Conclusions

This single-center prospective study provides important insights into the outcomes of transcatheter RVOT interventions in neonates and infants. BPV is effective in relieving isolated pulmonary stenosis, though outcomes are feasible and associated with favourable short-term physiology. RVOT perforation with BPV and PDA stenting shows good efficacy in PA-IVS, promoting pulmonary artery growth and enabling biventricular repair in the majority. RVOT stenting offers a reliable palliative approach for TOF and DORV physiology, supporting pulmonary artery development and facilitating later definitive repair. These findings underscore the importance of tailored intervention strategies and highlight the need for long-term monitoring and further research.
