# Correction: Immediate and Intermediate-Term Outcomes of Right Ventricular Outflow Tract Interventions in Neonates and Infants: A Prospective Single-Center Study

**DOI:** 10.7759/cureus.c491

**Published:** 2026-10-13

**Authors:** Bhavik Champaneri, Vicky Garhwal, Abhay Pota, Tarun Parmar, Shilpa Deodhar, Amit Kungwani

**Affiliations:** 1 Pediatric Cardiology, U.N. Mehta Institute of Cardiology and Research Centre, Ahmedabad, IND; 2 Cardiology Department, Geetanjali Medical College and Hospital, Udaipur, IND; 3 Anesthesia, U.N. Mehta Institute of Cardiology and Research Centre, Ahmedabad, IND

The affiliation for Dr. Vicky Garhwal has been changed to: Cardiology Department, Geetanjali Medical College and Hospital, Udaipur, India.

